# Characterization of Two Rhs-Family Nuclease Toxins, YPO3609 and YPO3615, in *Yersinia pestis*

**DOI:** 10.3390/microorganisms14081646

**Published:** 2026-07-28

**Authors:** Ran Duan, Yu Han, Yali Su, Wei Li, Kunkun Wang, Saisen Ji, Yue Xiao, Yuanming Huang, Huaiqi Jing, Xin Wang, Biao Kan, Weili Liang

**Affiliations:** National Key Laboratory of Intelligent Tracking and Forecasting for Infectious Diseases, National Institute for Communicable Disease Control and Prevention, Chinese Center for Disease Control and Prevention & Chinese Academy of Preventive Medicine, Beijing 102206, China; duanran@icdc.cn (R.D.); hanyu@sdmpu.edu.cn (Y.H.); 15881690607@163.com (Y.S.); liwei_9105@163.com (W.L.); kkwang1212@163.com (K.W.); 13376836864@163.com (S.J.); xiaoyue@icdc.cn (Y.X.); huangyuanming@icdc.cn (Y.H.); jinghuaiqi@icdc.cn (H.J.); wangxin@icdc.cn (X.W.); kanbiao@icdc.cn (B.K.)

**Keywords:** *Yersinia pestis*, Rhs toxin, nuclease activity, cognate immunity protein, polymorphic toxin system

## Abstract

Plague, caused by *Yersinia pestis*, remains a significant public health concern, yet the determinants of many toxin-associated proteins encoded by this pathogen remain poorly understood. Here, we characterized two previously unexplored Rearrangement hotspot (Rhs) proteins, YPO3609 and YPO3615, using a combination of bioinformatic analyses, bacterial toxicity assays, DNA degradation assays, SOS reporter assays, and RT-qPCR analysis. The C-terminal domains of YPO3609 and YPO3615 confer nuclease toxicity, with YPO3609 containing a WHH motif of the HNH nuclease superfamily and YPO3615 harboring a restriction endonuclease-like aspartate dyad. Heterologous expression of these C-terminal toxin domains in *Escherichia coli* inhibited growth, caused DNA degradation, elicited SOS-related responses, and induced cellular filamentation. Co-expression experiments identified YPO3610 and YPO3616 as the cognate immunity proteins of YPO3609 and YPO3615, respectively, while site-directed mutagenesis further showed that H411 and D1442/D1447 contribute to the nuclease-associated activities of the two toxins. Homologs of both toxin domains were broadly distributed in Pseudomonadota, with the C-terminal domain of YPO3609 showing a wider distribution. These findings characterize two functional Rhs toxin–immunity modules encoded by *Y. pestis*, and provide new insights into Rhs-associated nuclease toxins and their related homologs.

## 1. Introduction

Plague is a severe infectious disease caused by *Yersinia pestis* (*Y. pestis*) and has caused three major historical pandemics, with some estimates suggesting up to 200 million deaths throughout recorded history [1]. *Y. pestis* is naturally maintained in a rodent–flea enzootic cycle, with spillover to humans. Its persistence and transmission require adaptation to distinct biological niches, including survival in flea vectors and infection of animal hosts [2]. Although several determinants required for flea colonization and transmission, including *ymt* and the *hms* biofilm system [3], and metal acquisition systems important for mammalian infection, such as yersiniabactin and ZnuABC [4], have been identified in *Y. pestis*, the functions of many toxin-associated proteins encoded by this pathogen remain incompletely characterized.

The polymorphic toxin system (PTS) is distributed across diverse bacterial lineages and is commonly associated with interbacterial antagonism. These systems typically comprise a multidomain toxin carrying a variable C-terminal domain, a cognate immunity protein, and multiple cassettes encoding alternative toxic domains. The C-terminal region of polymorphic toxins generally confers the toxic activity, whereas the N-terminal region is involved in trafficking or delivery [5]. Positioned directly downstream of toxin genes, immunity genes encode small, highly specific proteins that protect producer cells from intoxication by their own toxins or by toxins delivered by neighboring cells. PTS comprise several well-characterized antibacterial toxin classes, such as colicins and pyocins [6], contact-dependent growth inhibition (CDI) systems [7], Rearrangement hotspot (Rhs) toxins [8], and some type VI secretion system (T6SS) effectors [9].

Among these systems, Rhs proteins represent one of the most prominent classes of polymorphic toxins. They are characterized by a conserved Rhs core region followed by a variable C-terminal toxin domain. The C-terminal boundary of the conserved Rhs core was originally defined by a highly conserved 61 amino-acid motif terminating in a bipartite DPxG-x_18_-DPxG sequence [10]. In Enterobacteriaceae, the immediate boundary of the Rhs-CT region is more commonly described by the highly conserved PxxxxDPxGL motif, which marks the transition from the conserved Rhs core/barrel to the polymorphic C-terminal toxin domain [11]. The C-terminal domains encode diverse toxic activities, including nucleases, proteases, and ADP-ribosyltransferases, with nucleases being particularly common. These nucleases can target DNA, RNA, or specific tRNAs, thereby disrupting genetic information flow and protein synthesis in intoxicated cells and rapidly inhibiting the growth of competing bacteria [8,12]. Previous genome sequencing revealed that *Y. pestis* contains genes encoding large Rhs-associated proteins characterized by repeat peptide motifs [13]. However, the biological functions of these loci in *Y. pestis* remain largely unexplored.

In this study, we identified two uncharacterized Rhs-family proteins in *Y. pestis*, YPO3609 and YPO3615, which exert antibacterial activity through their C-terminal nuclease domains. YPO3610 and YPO3616 are the corresponding immunity proteins conferring neutralization effects. Homologs of the C-terminal domains of YPO3609 and YPO3615 are predominantly distributed among members of the phylum Pseudomonadota. Together, this work provides a basis for understanding the nuclease-associated activities and immunity functions of these *Y. pestis*-encoded Rhs toxin–immunity modules.

## 2. Materials and Methods

### 2.1. Bacterial Strains and Culture Conditions

The strains and plasmids, including constructed plasmids, used in this study are listed in Table A1. *Y. pestis* strain EV76, the live-attenuated vaccine strain, was cultured in Luria–Bertani (LB) broth at 26 °C. *Escherichia coli* (*E. coli*) strains DH5α, MG1655, and BL21(DE3) were routinely cultured on LB agar plates or in LB broth at 37 °C, where indicated. Media were supplemented with ampicillin (Amp, 100 µg/mL), chloramphenicol (Cm, 10 µg/mL), 0.2% or 0.02% (*w*/*v*) L-arabinose, and 0.2% (*w*/*v*) D-glucose, ciprofloxacin (CIP, 0.1 μg/mL), or mitomycin C (MMC, 0.5 μg/mL).

### 2.2. Plasmid Construction and Site-Directed Mutagenesis

Genomic DNA from *Y. pestis* EV76 was used as a template. To assess the potential toxicity of YPO3609 and YPO3615, the coding sequences of the C-terminal regions of YPO3609 (residues 306–456) and YPO3615 (residues 1382–1512) were respectively amplified with primer pairs EcoRI-3609CT-p24F/EV3609-F2dn and KpnI-3615(1382)-p33F/3615CT-F2dn (Table A2) and cloned into pBAD vectors. To evaluate the protective effects of the cognate immunity proteins, the coding sequences of YPO3610 and YPO3616 were amplified using primer pairs KpnI-3610-pGHF/HindIII-3610-pGHR and EcoRI-3616-p24F/HindIII-3616-p24R, respectively, and subsequently cloned into compatible pBAD vectors.

Site-directed mutagenesis was performed by overlap PCR. The mutant construct encoding YPO3609^306–456(H411A)^ was generated using primer pairs EcoRI-3609CT-p24F/EV3609-H411A-F1dn and EV3609-H411A-F2up/EV3609-F2dn, and then cloned into pBAD24 to yield pBAD24-YPO3609^306–456(H411A)^. The double mutant variant encoding YPO3615^1382–1512(D1442N, D1447N)^ was created with primer pairs KpnI-3615(1382)-p33F/3615CT-DD-F1dn and 3615CT-DD-F2up/3615CT-F2dn, and subsequently cloned into pBAD33 to generate pBAD33-YPO3615^1382–1512(D1442N, D1447N)^. The primers used are listed in Table A2.

All recombinant plasmids (Table A1) were constructed in DH5α and verified by DNA sequencing. The verified plasmids were then introduced into MG1655 by heat-shock transformation using chemically competent cells prepared with Competent Cell Preparation Kit (TaKaRa, Dalian, China), and the resulting transformants were used for the bacterial toxicity assay. During plasmid construction and maintenance, 0.2% (*w*/*v*) D-glucose was included to repress basal expression from the pBAD promoter.

For the SOS reporter test, the promoter region of the *Y. pestis recA* gene was amplified with primer pair PrecA500-SacI-F/PrecA-BamHI-rev and cloned into the pBBR*lux* plasmid to generate pBBR*lux-recA*, which served as the SOS reporter and was transformed into *E. coli* BL21 (DE3).

### 2.3. Bacterial Toxicity Assays and Microscopic Examination

*E. coli* cells carrying the indicated constructs were harvested from overnight LB agar plate cultures and resuspended in LB broth, and the bacterial density was adjusted to 3.5 McFarland (McF) units using a densitometer (bioMérieux, Marcy-l'Étoile, France). The resulting cell suspension was subsequently diluted 1:100 in fresh LB broth and incubated at 37 °C for 1 h with shaking. The cultures were then divided equally into two aliquots. One aliquot was induced with 0.2% (*w*/*v*) L-arabinose, whereas the other was supplemented with 0.2% (*w*/*v*) D-glucose as a repression control. The cultures were further incubated at 37 °C for 3 h. Aliquots of cultures (40 μL) were serially diluted 10-fold, and 10 μL of each dilution was spotted onto LB agar plates with appropriate antibiotics. The plates were then incubated overnight at 37 °C. For microscopic examination, 5 μL of the culture was subjected to Gram staining and examined using a microscope (Nikon 80i, Tokyo, Japan). Images were captured using a 100× oil-immersion objective, with focus maintained by the IS-Elements F3.2.

### 2.4. DNA Degradation Assay

The *E. coli* MG1655 strain carrying the indicated constructs was cultured as described above for the bacterial toxicity assay, except that a larger culture volume (15 mL) was used. After 3-h incubation under induced or repressed conditions, bacterial pellets were collected, resuspended in LB broth, and normalized to 2.5 McF. Equal volumes of normalized cell cultures were then used for separate extraction of plasmid DNA or genomic DNA (Tiangen Biotech, Beijing, China). Nucleic acid concentrations were measured using a NanoDrop spectrophotometer (Thermo Fisher Scientific, Waltham, MA, USA), and equal amounts of DNA were analyzed by electrophoresis on a 1% agarose gel.

### 2.5. Luminescence Activity Assay

The effect of the wild-type (WT) or mutant C-terminal toxin regions of YPO3609 or YPO3615 on *recA* promoter activity was quantified using the pBBR*lux-recA*/BL21(DE3) reporter strain. Briefly, this reporter strain was separately transformed with the indicated L-arabinose-inducible pBAD-derived plasmids expressing the WT or mutant C-terminal toxin domains, or with the empty pBAD24 vector as a negative control. Cells carrying the empty pBAD24 vector treated with CIP (0.1 μg/mL) [14] or MMC (0.5 μg/mL) [15] served as positive controls, respectively. *E. coli* cells from overnight LB agar plate cultures were resuspended in LB broth, adjusted to 6.5 McF, diluted 1:100 into fresh LB broth containing 0.02% (*w*/*v*) L-arabinose, and incubated at 37 °C with shaking. Luminescence and OD_600_ were measured by transferring 200 μL of each culture into a 96-well microplate (Corning, Corning, NY, USA) and read using a microplate reader (PerkinElmer, Waltham, MA, USA). Levels of *recA* promoter activity were expressed as luminescence normalized to cell density (Lux/OD_600_).

### 2.6. RNA Extraction and Quantitative Real-Time PCR

Following the 3-h incubation as detailed in the DNA degradation assay, cultures were collected and resuspended in 1 mL of fresh LB broth and adjusted to 2.5 McF. Total RNA extraction and cDNA synthesis were performed as previously described [16]. The equation R = 2^- (Cq target -Cq reference)^ was used to calculate the relative expression values (R), where Cq is the quantification cycle. The gene *rpoD*, previously used in bacterial qRT-PCR analyses under DNA damage- or SOS-related conditions [17,18,19], was used as the reference gene to normalize the mRNA expression levels of *sulA* [20] and *ftsZ* [21] (Table A2). For each sample, DNase-treated RNA without reverse transcription was used as a negative control to rule out genomic DNA contamination.

### 2.7. Sequence Feature and Homolog Identification

The domain architecture and conserved motifs of YPO3609 and YPO3615 were analyzed as follows. Potential functional domains were searched using the NCBI Conserved Domain Database (CDD) [22] and the HHpred server [23]. Multiple sequence alignments of amino acid sequences were generated in MEGA X [24], and the sequence conservation logo was created with WebLogo 3 [25] (http://weblogo.threeplusone.com/). Domain architectures were illustrated with IBS 2.0 [26]. The potential effects of amino acid substitutions on protein stability were predicted using DUET [27] and DynaMut2 [28], based on the predicted changes in folding free energy (ΔΔG).

Proteins containing sequences homologous to C-terminal regions of YPO3609 and YPO3615 (designated YPO3609CT and YPO3615CT, respectively) were identified using BLASTp [29]. BLASTp hits were filtered based on the following thresholds: sequence identity of no less than 30%, query coverage of at least 50%, and an E-value of 10^−9^ or lower. To exclude truncated sequences, proteins shorter than the corresponding query C-terminal regions were removed: proteins shorter than 151 amino acids (aa) were excluded from the YPO3609CT homolog set, and those shorter than 135 aa were excluded from the YPO3615CT homolog set. Taxonomic assignments were determined based on the NCBI Taxonomy database [30], and distributions across taxonomic ranks, from phylum to species, were visualized using SankeyMATIC (http://sankeymatic.com/).

Conserved domains in the retrieved homologs were further analyzed by scanning against profile hidden Markov models (profile-HMMs) using HMMER (hmmscan) [31,32]. The profile-HMMs associated with Rhs repeats and DUF6531 were obtained from Pfam (PF03527: RHS; PF05593: RHS_repeat; PF20148: DUF6531) [33] and TIGRFAM (TIGR03696: Rhs_assc_core/RAC) [34]. Homologs were designated as Rhs-positive if at least one Rhs-associated HMM profile was identified in either database; otherwise, they were classified as Rhs-negative. For Rhs-negative homologs, the upstream sequences of the C-terminal homologous regions were further scanned against Pfam-A (https://www.ebi.ac.uk/interpro/download/pfam/; accessed on 17 December 2025) to identify potential associated domains.

### 2.8. Genomic Context Extraction and Comparative Analysis

Whole genomes containing YPO3609CT-YPO3610 or YPO3615CT-YPO3616 homolog pairs were identified by tBLASTn searches. Genomic regions encompassing each homolog and its flanking sequences (±40 kb) were extracted using seqkit (v2.2.0) [35]. The extracted sequences were subsequently annotated using Bakta (v1.9.4) [36] with default parameters. Representative genomic regions were selected for downstream comparative analysis, and gene cluster organization was visualized using clinker (v0.0.32) [37].

## 3. Result

### 3.1. Bioinformatic Analysis of Two Potential Rhs Nuclease-Cognate Immunity Pairs

**Figure 1:**
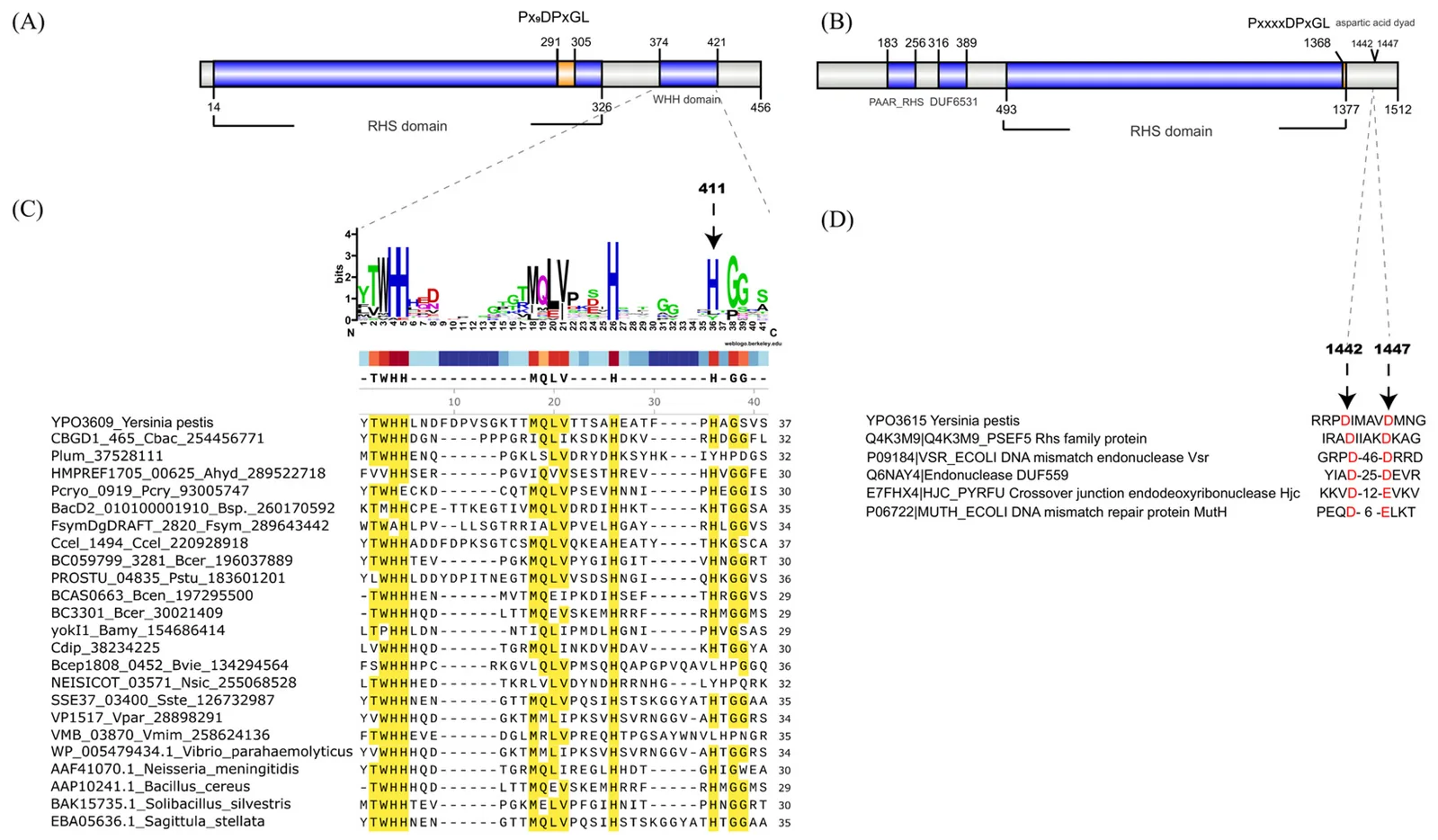
Structural domain analysis and conserved amino acid site identification of two putative Rhs proteins. Schematic representation of the domain architectures of the YPO3609 protein (**A**) and the YPO3615 protein (**B**). The predicted domains are marked in blue, the Px_9_DPxGL or PxxxxDPxGL motif within the Rhs domain is highlighted in orange, and the remaining regions are shown in gray. (**C**) Multiple amino acid sequence alignment of WHH domains from YPO3609 and representative WHH nucleases. Amino acid residues at each position are depicted as a sequence logo, with larger symbols indicating higher conservation. Red and blue color blocks below indicate conserved and variable sites, respectively, with conserved sites marked by a yellow background behind the letters. The arrow indicates the selected H411 residue for alanine substitution. (**D**) Multiple amino acid sequence alignment of the C-terminal restriction endonuclease-like aspartate dyad region of YPO3615 with representative homologous regions. The conserved aspartate residues targeted for asparagine substitution are indicated by arrows.

Through bioinformatic analysis, we identified two putative Rhs toxin–immunity pairs, YPO3609-YPO3610 and YPO3615-YPO3616. Although YPO3609 and YPO3615 have been annotated as Rhs-associated proteins [38,39], their functions as Rhs-family nuclease toxins had not been experimentally characterized. Here, we demonstrate that both function as Rhs-family nuclease toxins and are neutralized by their cognate immunity proteins. Sequence analysis showed that YPO3609 is 456 aa in length and contains a Rhs-repeat domain (residues 14–326) and a C-terminal WHH domain (residues 374–421) (Figure 1A). Within the Rhs domain, a well-conserved Px_9_DPxGL motif (residues 291–305) was identified [40], which serves as a characteristic linker between the core Rhs region and the variable C-terminal toxin module in polymorphic toxin systems. Accordingly, the C-terminal region following the Rhs core represents a variable toxin module harboring a WHH domain. The WHH domain has been documented as a minimal functional module belonging to the HNH nuclease superfamily [41]. Multiple sequence alignment of the WHH domain with those of known nucleases showed that it retains the conserved signature motif, comprising one tryptophan (W) and four histidine (H) residues, characteristic of functional WHH domains (Figure 1C). Based on the study of WHH toxin RhsP in *Vibrio parahaemolyticus*, mutation of the fourth H (RhsP^H1354A^) can lead to the loss of nuclease activity [42]. H411 represents the corresponding fourth histidine in the WHH domain of YPO3609; it was selected for subsequent site-directed mutagenesis to validate its functional contribution to the toxicity of YPO3609. The ypo3610 gene is located downstream of ypo3609 and encodes an SMI1/KNR4 (SUKH-1) family protein. SMI1 proteins are primary immunity proteins in bacterial toxin systems [41]. Therefore, we hypothesized that YPO3610 may be an immunity protein for YPO3609. Collectively, these data indicate that YPO3609-YPO3610 is a putative Rhs toxin–immunity pair.

YPO3615 has a total length of 1512 residues and possesses a PAAR_RHS domain (residues 183–256), a DUF6531 domain (residues 316–389), and a Rhs domain (residues 493–1377) (Figure 1B). Similar to YPO3609, the Rhs domain of YPO3615 contains a PxxxxDPxGL motif (residues 1368–1377). Multiple sequence alignment of its C-terminal region with other nucleases suggested that D1442 and D1447 constitute active aspartic acid dyads, which are conserved functional sites in the restriction enzyme-related superfamily (Figure 1D) [43]. The ypo3616 gene is located downstream of ypo3615 and encodes a hypothetical protein with a predicted coiled-coil region (residues 43–70). Based on the conserved toxin–immunity gene organization, we proposed that YPO3616 is the immunity protein of YPO3615.

### 3.2. YPO3609 and YPO3615 Inhibit the Growth of E. coli

**Figure 2:**
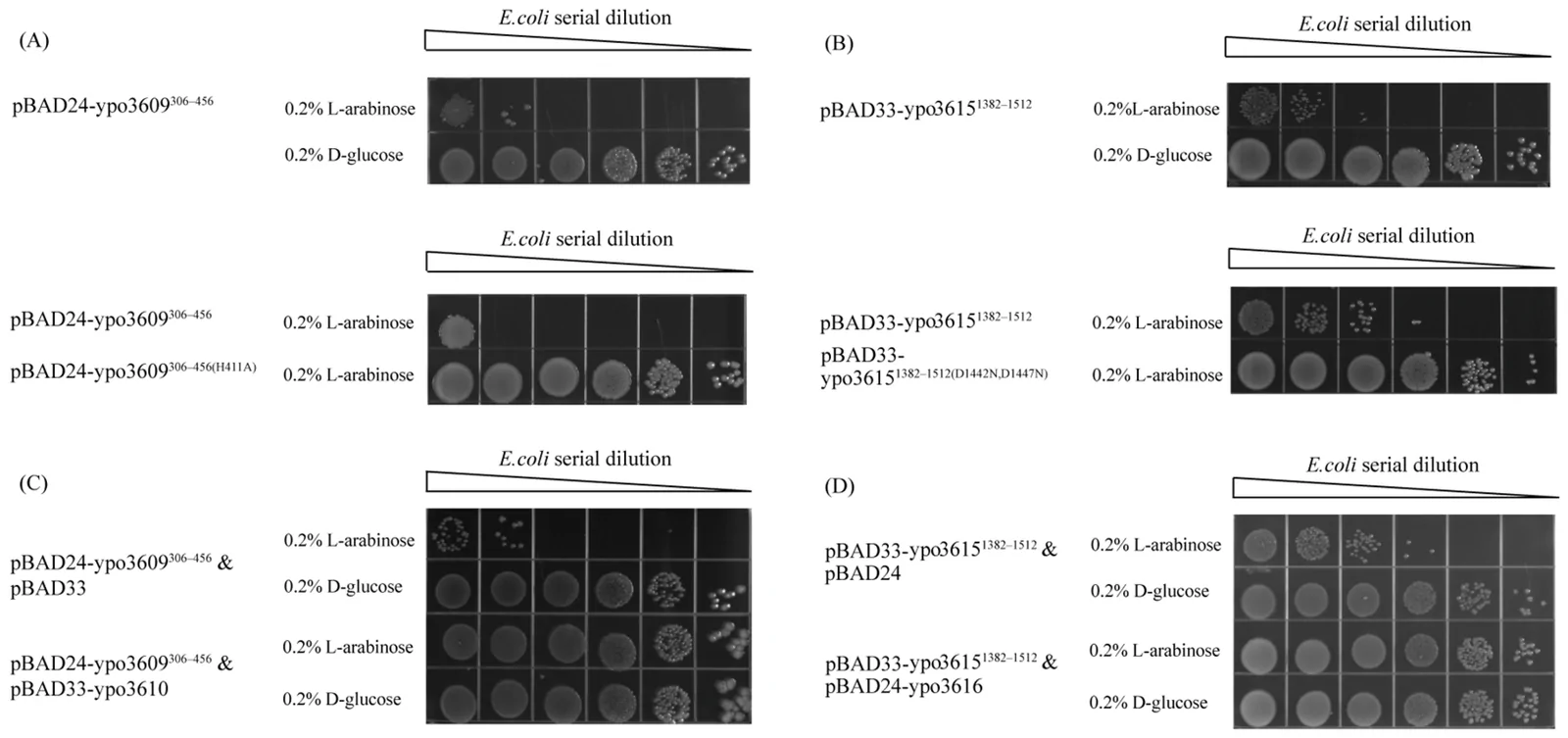
YPO3609-YPO3610 and YPO3615-YPO3616 are antibacterial toxin–immunity pairs. (**A**,**B**) Antibacterial activities of the C-terminal regions of YPO3609 (A), YPO3615 (**B**), and their variants with selected residue substitutions. (**C**,**D**) Neutralization of toxicity by cognate immunity proteins. Survival of *E. coli* MG1655 co-expressing YPO3609 C-terminal and YPO3610 (**C**) or YPO3615 C-terminal and YPO3616 (**D**). *E. coli* MG1655 harboring the indicated recombinant plasmids was induced by 0.2% L-arabinose or repressed by 0.2% D-glucose for 3 h in LB broth. Bacterial survival was assessed by plating serial dilutions.

To verify whether YPO3609 and YPO3615 possess antibacterial activity, we intended to heterologously express them in *E. coli* MG1655. Attempts to construct full-length expression plasmids were unsuccessful even with 0.2% D-glucose repression. Therefore, we constructed C-terminal expression plasmids, pBAD24-ypo3609^306–456^ and pBAD33-ypo3615^1382–1512^. Expression of these truncations was repressed by 0.2% D-glucose and activated by 0.2% L-arabinose in MG1655. As shown in Figure 2, no killing effect was observed in the D-glucose-repressed groups, whereas a significant decrease in colony-forming units (CFUs) was observed in the L-arabinose-induced groups, indicating that both YPO3609 and YPO3615 exhibit toxicity toward *E. coli.* (Figure 2A,B).

As described above, the C-terminal WHH domain of YPO3609 contains a conserved tryptophan (W) residue and four histidine (H) residues. To validate the functional significance of the WHH domain for YPO3609 antibacterial activity, we introduced a histidine-to-alanine substitution at residue 411 (H411A) into the construct expressing the C-terminal region of YPO3609, generating pBAD24-ypo3609^306–456 (H411A)^. After induction with 0.2% L-arabinose, this mutant showed markedly reduced toxicity toward *E. coli* MG1655, similar to the level observed in the 0.2% D-glucose-repressed WT group, supporting the functional importance of the conserved H residue (H411) in the WHH domain for the antibacterial activity of the YPO3609 C-terminus (Figure 2A). For YPO3615, we introduced aspartate-to-asparagine substitutions at residues 1442 and 1447 (D1442N and D1447N) into the construct pBAD33-ypo3615^1382–1512^ to generate pBAD33-ypo3615^1382–1512 (D1442N, D1447N)^. Unlike the WT construct, this mutant exhibited markedly attenuated antibacterial activity upon induction (Figure 2B), supporting the functional importance of D1442 and D1447 residues in the C-terminal region of YPO3615.

To assess whether the reduced toxicity of the mutants could be related to altered protein stability, we evaluated the potential effects of these substitutions using DUET and DynaMut2. The H411A mutation in YPO3609^306–456^ was predicted to be stabilizing by both tools (ΔΔG values = +0.521 kcal/mol by DUET and +1.05 kcal/mol by DynaMut2). For YPO3615^1382–1512^, the D1442N and D1447N mutations were predicted to be destabilizing (ΔΔG = −0.752 and −1.69 kcal/mol by DUET; −0.77 and −1.38 kcal/mol by DynaMut2). These predictions suggest that the attenuated phenotype of YPO3609^306–456 (H411A)^ is less likely to be explained by protein destabilization, whereas reduced stability may contribute to the phenotype of the YPO3615^1382–1512 (D1442N, D1447N)^ mutant.

To test whether the downstream genes ypo3610 and ypo3616 encode immunity proteins, we constructed recombinant plasmids pBAD33-ypo3610 and pBAD24-ypo3616 expressing the respective potential immunity protein. These were co-expressed with their respective toxin C-terminal recombinant plasmids in *E. coli*. When co-expressed with the empty vector pBAD33, the YPO3609^306–456^ toxin protein showed significant antibacterial activity upon induction (Figure 2C). However, co-expression of YPO3610 from the plasmid pBAD33-ypo3610 significantly reduced this toxicity, indicating that YPO3610 antagonizes the antibacterial effect of YPO3609^306–456^ (Figure 2C). Similarly, co-expression of YPO3616 efficiently neutralized the toxicity of YPO3615^1382–1512^ (Figure 2D). Together, these results demonstrate that YPO3610 and YPO3616 are the cognate immunity proteins for YPO3609 and YPO3615, respectively.

### 3.3. YPO3609 and YPO3615 Induce DNA Degradation

**Figure 3:**
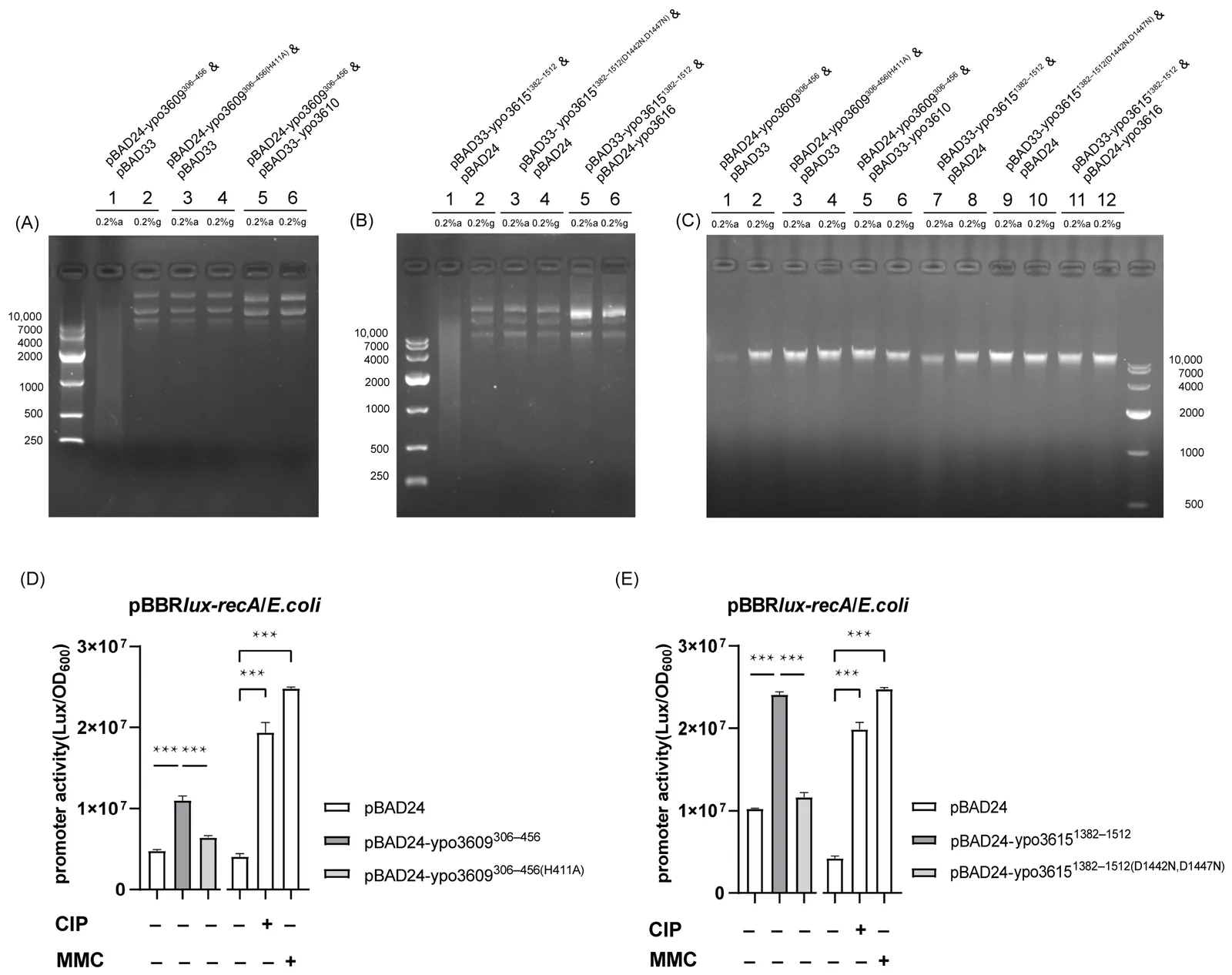
Nuclease activity assays and effects of YPO3609^306–456^ and YPO3615^1382–1512^ on *recA* promoter activity. (**A**) Plasmid extracted from *E. coli* harboring the indicated recombinant plasmid expressing YPO3609^306–456^ or its mutant YPO3609^306–456 (H411A)^, alongside the empty control vector or the YPO3610 immunity expression construct. (**B**) Plasmid extracted from *E. coli* containing the indicated construct expressing YPO3615^1382–1512^ or YPO3615^1382–1512 (D1442N, D1447N)^ together with the control vector or the YPO3616 immunity construct. (**C**) Purified genomic DNA from *E. coli* with the indicated plasmids; 0.2%a indicates addition of 0.2% L-arabinose, and 0.2%g indicates 0.2% D-glucose. DNA was visualized in agarose gels containing GelStain nucleic acid dye, and the DNA marker was labeled on the side of each Figure. (**D**,**E**) Levels of r*ecA* promoter activity by YPO3609^306–456^, YPO3615^1382–1512^, and their respective mutant variants. The activity was measured using a *recA* promoter-driven luminescence reporter pBBR*lux-recA* in *E. coli* BL21 (DE3). The empty vector pBAD24 served as a negative control, whereas pBAD24-bearing cells treated with CIP (0.1 μg/mL) or MMC (0.5 μg/mL) served as positive controls. Cultures were induced with 0.02% L-arabinose, and luminescence was measured at the 4-h incubation time point. Data are shown as mean ± SD (n = 3), ***, *p* < 0.001.

To investigate the nuclease activity of YPO3609 and YPO3615, we performed DNA degradation assays in *E. coli*. For YPO3609, induction of the pBAD24-ypo3609^306–456^ construct with 0.2% L-arabinose triggered robust degradation of plasmid DNA (Figure 3A, lane 1) and chromosomal DNA isolated from *E. coli* (Figure 3C, lane 1), whereas repression with 0.2% D-glucose showed no degradation (Figure 3A, lane 2; Figure 3C, lane 2), indicating that the C-terminal region possesses nuclease activity. To assess the contribution of the conserved histidine residue to nuclease activity, the pBAD24-ypo3609^306–456 (H411A)^ construct was used. As expected, the H411A mutant showed markedly reduced DNA degradation of both the plasmid and chromosomal DNA (Figure 3A,C, lane 3), supporting the functional importance of H411 as a conserved putative active-site residue in YPO3609 [42]. Finally, to test immunity protection, YPO3610 was co-expressed with pBAD24-ypo3609^306–456^. Co-expression of YPO3610 neutralized the YPO3609^306–456^-induced DNA breakdown (Figure 3A, lane 5; Figure 3C, lane 5), and no significant difference was observed between the L-arabinose-induced and D-glucose-repressed conditions (Figure 3A,C, lanes 5–6). Thus, YPO3609 exerts nuclease-associated toxicity, while YPO3610, as a cognate immunity protein, antagonizes its activity.

Similarly, for YPO3615, induction of the construct pBAD33-ypo3615^1382–1512^ producing its C-terminal domain with L-arabinose caused marked degradation of both plasmid and genomic DNA (Figure 3B, lane 1; Figure 3C, lane 7). Then, the mutant construct carrying substitutions in the conserved aspartate dyad, pBAD33-ypo3615^1382–1512 (D1442N, D1447N)^, was examined. Upon induction, DNA degradation was markedly attenuated (Figure 3B, lane 3; Figure 3C, lane 9). Mutation of D1442 and D1447 attenuated the nuclease-associated phenotype, supporting their roles as putative functional residues in the C-terminal region of YPO3615. In addition, co-expression of YPO3616 neutralized the nuclease effect of YPO3615^1382–1512^ (Figure 3B, lane 5; Figure 3C, lane 11). Hence, these results support that YPO3616 acts as an immunity protein to antagonize the nuclease activity of YPO3615. In summary, YPO3609 and YPO3615 exert nuclease activity through their C-terminal WHH domain and restriction endonuclease-like domain, respectively. YPO3610 and YPO3616 function as cognate immunity proteins neutralizing the nuclease activity of their respective toxin partners.

### 3.4. YPO3609 and YPO3615 Induce the SOS-Related Response

The bacterial SOS response is a global regulatory network triggered by DNA damage [44]. To determine whether YPO3609 and YPO3615 elicit SOS-related responses, we monitored *recA* promoter activity, a key marker of SOS induction. The promoter region of *recA* from *Y. pestis* was cloned into the promoterless reporter plasmid pBBR*lux*, and the resulting recombinant plasmid pBBR*lux*-*recA* was introduced into *E. coli* BL21 (DE3) to generate a heterologous SOS response reporter strain, pBBR*lux-recA*/BL21 (DE3). The responsiveness of this reporter system was verified using the DNA-damaging agent ciprofloxacin (CIP) and mitomycin C (MMC) as positive controls. In reporter cells carrying the empty pBAD24 vector, CIP and MMC increased *recA* promoter activity by approximately 4.8-fold and 6.0-fold, respectively, compared with the corresponding untreated control, confirming that the reporter system responded effectively to established SOS-inducing agents (Figure 3D,E).

Under this validated reporter background, the induced expression of the C-terminal domains of the two toxin proteins significantly increased *recA* promoter activity in *Y. pestis.* Specifically, the *recA* promoter activity in the strain expressing YPO3609^306–456^ was 2.3-fold higher than that of the empty vector control (Figure 3D), and that of the strain expressing YPO3615^1382–1512^ was 2.4-fold higher than that of the empty vector control (Figure 3E). These results are consistent with the nuclease-associated activity of YPO3609 and YPO3615, suggesting that their C-terminal domains can cause DNA damage and induce SOS-related responses. In contrast, compared with the WT toxins, the *recA* promoter activity of the mutants YPO3609^306–456 (H411A)^ (Figure 3D) or YPO3615^1382–1512 (D1442N, D1447N)^ (Figure 3E) was markedly reduced to levels comparable to those of the empty vector group. These results support the functional importance of H411 as a conserved putative active-site histidine in the WHH domain of YPO3609, and identify D1442/D1447 as putative functional residues in YPO3615, respectively.

### 3.5. YPO3609 and YPO3615 Induce Filamentation of E. coli

**Figure 4:**
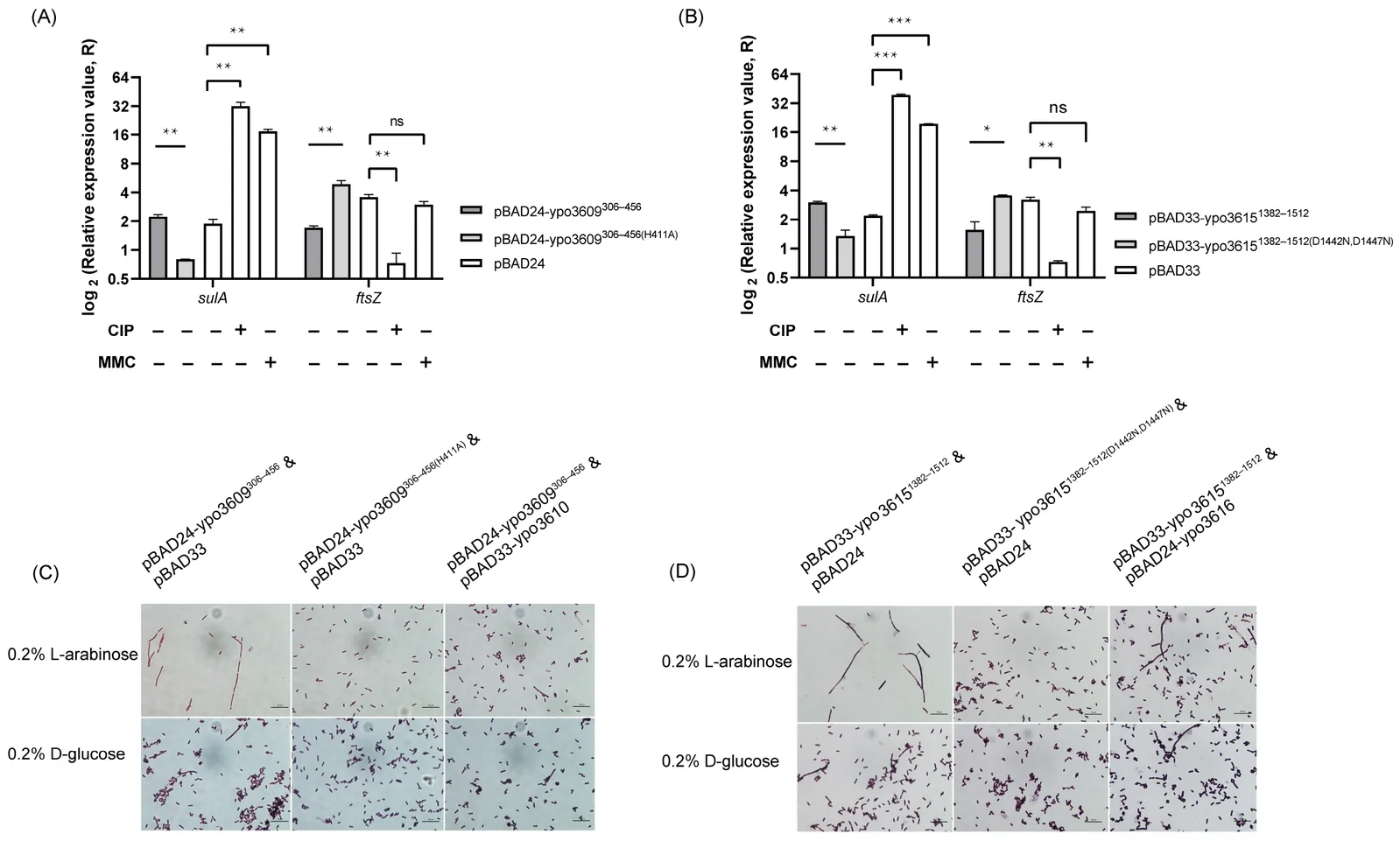
YPO3609^306–456^ and YPO3615^1382–1512^ induce bacterial filamentation**.** (**A**,**B**) The effects of YPO3609^306–456^ (**A**) and YPO3615^1382–1512^ (**B**) on the mRNA expression of *sulA* and *ftsZ* genes. pBAD24- or pBAD33-bearing cells treated with CIP (0.1 μg/mL) or MMC (0.5 μg/mL) served as positive controls. ns, not significant, *, *p* < 0.05, **, *p* < 0.01, ***, *p* < 0.001. Data are shown as mean ± SD (n = 3). (**C**,**D**) Gram-staining microscopy of *E. coli* harboring the indicated plasmids under induced or repressed conditions. (**C**) Cells expressing YPO3609^306–456^, its H411A mutant, or YPO3609^306–456^ together with its immunity protein YPO3610. (**D**) Cells expressing YPO3615^1382–1512^, its D1442N/D1447N mutant, or YPO3615^1382–1512^ along with its immunity protein YPO3616. Images were acquired using a 100× oil-immersion objective (1000 × total magnification).

One of the key SOS effector genes, *sulA*, encodes a cell division inhibitor that blocks FtsZ polymerization, thereby providing a plausible molecular basis for SOS-associated cell-division inhibition [45]. To determine whether the SOS-related responses observed upon expression of the two nucleases were accompanied by downstream effects on cell division, we examined the expression changes in these genes and bacterial morphology (Figure 4). CIP and MMC were included as positive controls and increased *sulA* expression by approximately 17.1- to 17.8-fold and 8.9- to 9.2-fold, respectively, confirming that the qRT-PCR assay reliably detected the induction of this canonical SOS gene (Figure 4A,B). For *ftsZ*, CIP reduced its expression to approximately 20–23% of the untreated control levels, whereas MMC caused no significant change, indicating variable transcriptional responses of *ftsZ* to different DNA-damaging agents. The *sulA* mRNA levels were significantly increased by approximately 2.8-fold and 2.3-fold in strains expressing the C-terminal regions of YPO3609 or YPO3615, respectively, compared with the corresponding mutant strains, YPO3609^306–456 (H411A)^ or YPO3615^1382–1512 (D1442N, D1447N)^ (Figure 4A,B). Microscopy revealed that *E. coli* expressing WT C-terminal toxin regions exhibited pronounced elongation and filamentous morphology under inducing conditions, whereas cells expressing the mutants appeared normal (Figure 4C,D). Together, these results indicate that the nuclease-active C-terminal toxin domains of YPO3609 and YPO3615 induce SOS-related transcriptional responses. Interestingly, we found that cell-division defect is also accompanied by reduced expression of the cell-division-related gene *ftsZ* (Figure 4A,B). This pattern differs from the canonical SOS response, in which cell division is mainly inhibited through SulA-mediated interference with FtsZ polymerization instead of direct repression of *ftsZ* transcription [46]. The reduced *ftsZ* mRNA levels observed here may reflect a secondary consequence of DNA damage-associated stress or growth inhibition, rather than the direct output of SOS regulation [47]. Furthermore, co-expression of the cognate immunity proteins YPO3610 or YPO3616 with the corresponding C-terminal toxins suppressed filamentation, further confirming the protective effects of these immunity proteins (Figure 4C,D).

### 3.6. Taxonomic Distribution of YPO3609CT and YPO3615CT Homologs and Genomic Contexts of Their Toxin–Immunity Module Pairs

**Figure A1:**
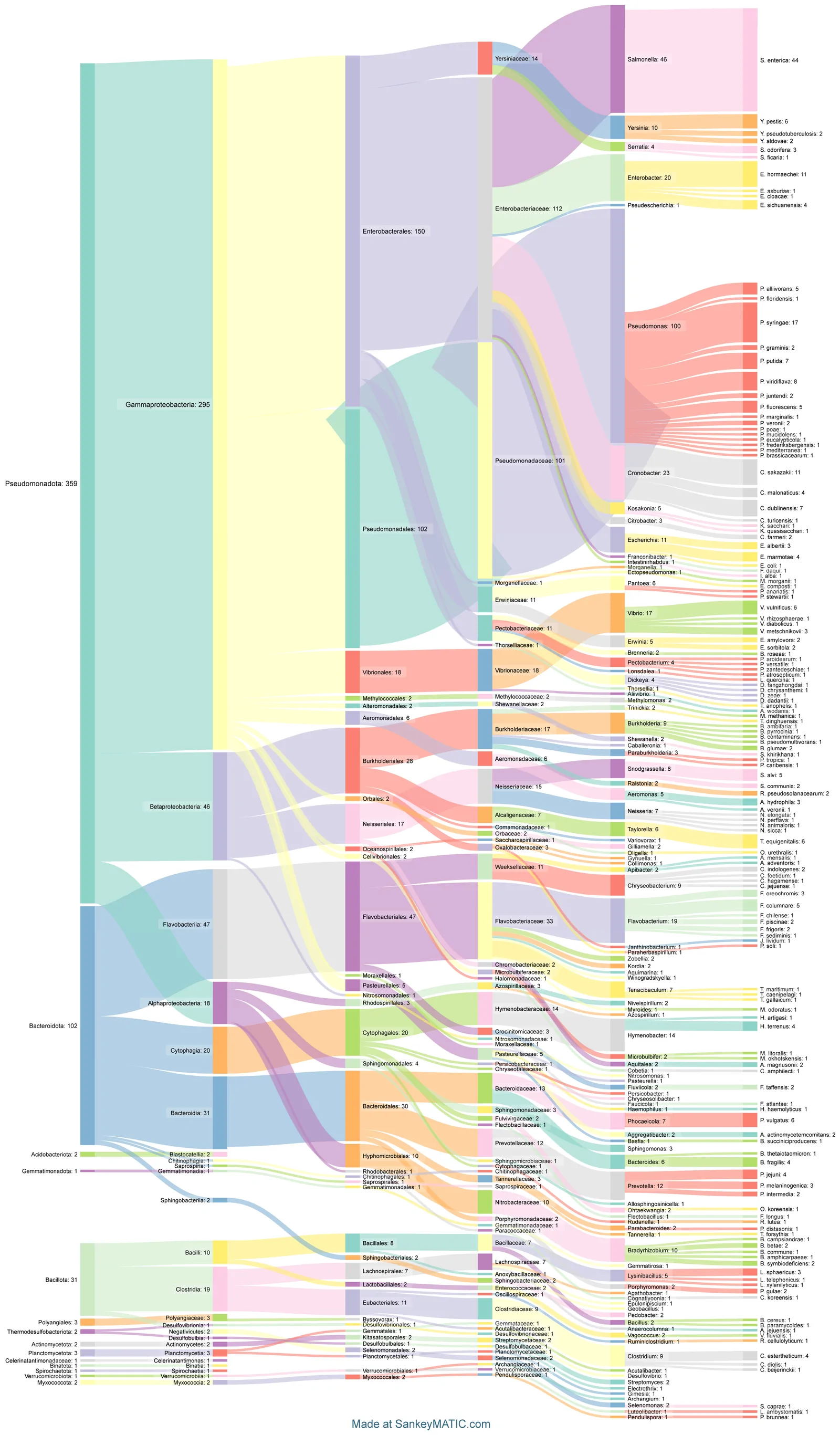
Taxonomic distribution analysis of YPO3609^306–456^ C-terminal domain. A Sankey diagram depicts the relationships between bacterial taxa (phylum, class, order, family, genus, and species from left to right). The number of sequences in each taxon is indicated.

**Figure A2:**
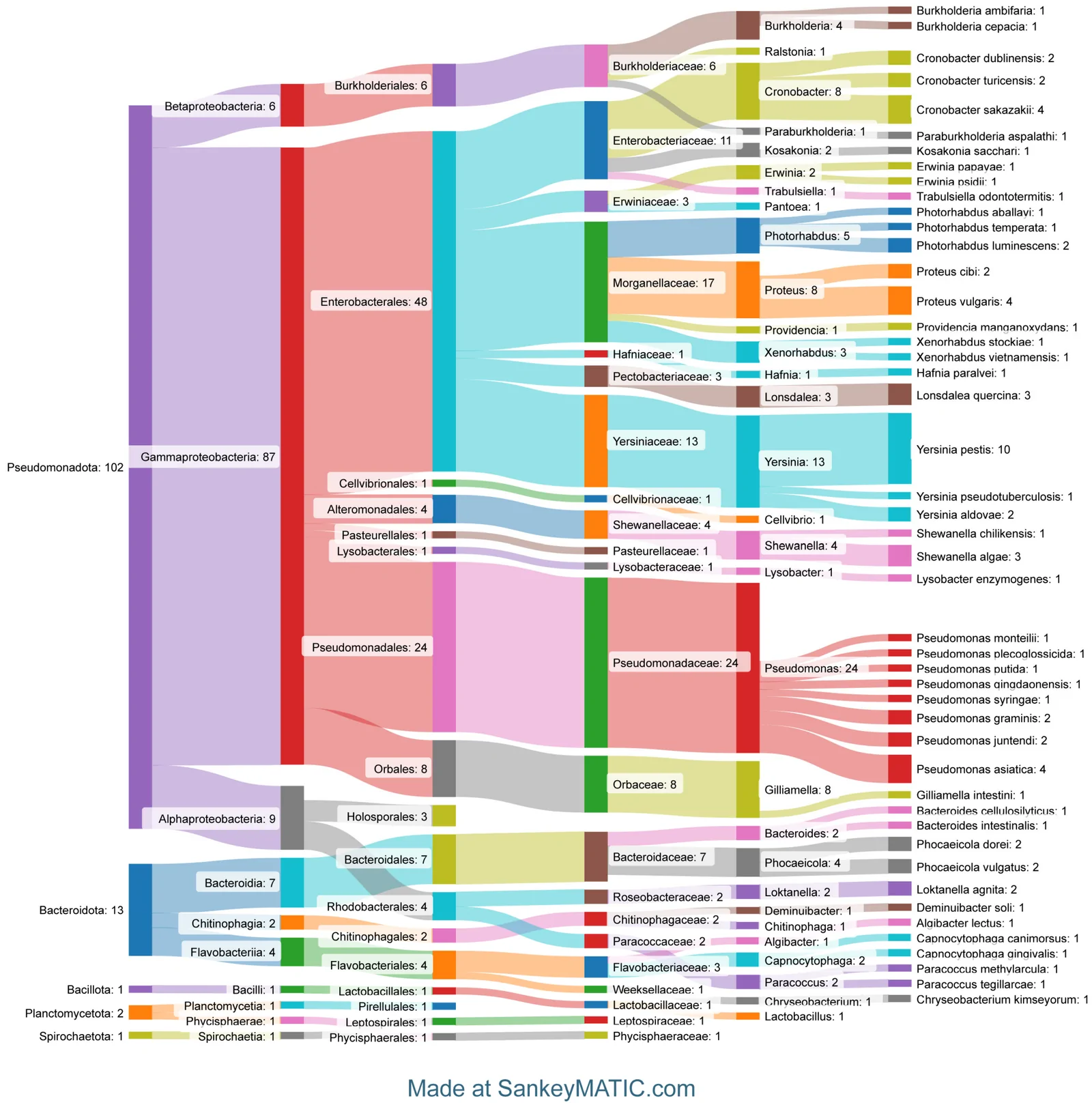
Taxonomic distribution analysis of YPO3615^1382–1512^ C-terminal domain. A Sankey diagram depicts the relationships between bacterial taxa (phylum, class, order, family, genus, and species from left to right). The number of sequences in each taxon is indicated.

Bioinformatic analysis revealed that homologs containing the C-terminal regions of YPO3609 and YPO3615 were both predominantly distributed within Pseudomonadota (70.2% and 83.6%, respectively), especially Gammaproteobacteria, but differed in the number, taxonomic breadth, and domain architectures of the identified homologs. A total of 513 YPO3609CT homologs were identified across 22 classes, 38 orders, 64 families, 100 genera, and 142 species (Figure A1), whereas 122 YPO3615CT homologs were identified across 10 classes, 17 orders, 22 families, 30 genera, and 47 species (Figure A2). Within Gammaproteobacteria, homologs of both toxin domains were mainly found in Enterobacterales and Pseudomonadales. Within Enterobacterales, YPO3609CT homologs were mainly distributed in Enterobacteriaceae and Yersiniaceae, whereas YPO3615CT homologs were mainly distributed in Morganellaceae and Yersiniaceae. In Pseudomonadales, homologs of both toxin domains were found predominantly in Pseudomonadaceae. Domain architecture analysis showed that most homologs were RHS-positive, accounting for 77.6% of YPO3609CT homologs and 87.7% of YPO3615CT homologs, while DUF6531 was detected only in Rhs-positive homologs. DUF6531 corresponds to the plug that seals the N-terminal aperture of the Rhs cocoon in T6SS-associated Rhs effectors [48]. Among Rhs-negative homologs, YPO3609CT homologs carried more diverse additional domains, including PT-HINT, DUF4280, TssD, WXG100 together with PT-TG, FN3, and LysM, whereas only two Rhs-negative YPO3615CT homologs carried Fil_haemagg_2 and DUF637, respectively.

**Figure 5:**
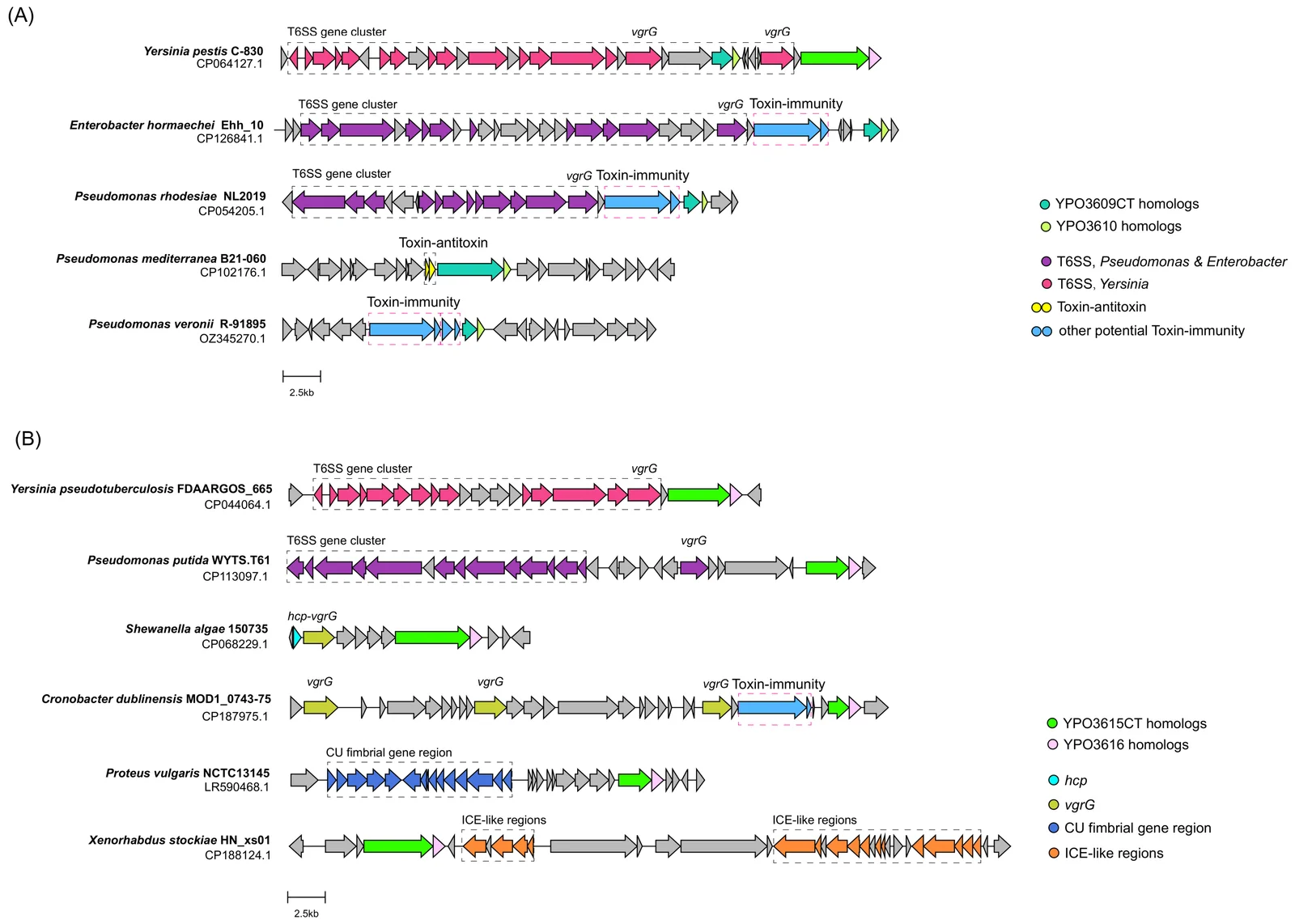
Representative genomic contexts of YPO3609CT-YPO3610 homologs (**A**) and YPO3615CT-YPO3616 homologs (**B**). The representative strains in each panel were selected from 457 and 282 genomes, respectively, harboring the respective toxin–immunity pairs, based on the diversity of genomic environments and taxonomic ranges. Different colors indicate distinct homologs and associated gene clusters, as shown in the legend. For T6SS loci, shared colors indicate similarity across species: red indicates *Yersinia* sp. T6SS clusters; purple indicates *Enterobacter* sp. and *Pseudomona*s sp. T6SS clusters. Dashed boxes indicate functionally related loci, and pink dashed boxes highlight the putative toxin–immunity modules.

To further examine the genomic contexts of these toxin–immunity modules, we analyzed 457 genomes harboring YPO3609CT-YPO3610 homolog pairs and 282 genomes harboring YPO3615CT-YPO3616 homolog pairs. Both module types were frequently found in T6SS-associated regions. For YPO3609CT-YPO3610, representative loci were observed in multiple *Yersinia*, *Enterobacter*, and *Pseudomonas* genomes (Figure 5A). For YPO3615CT-YPO3616, T6SS-associated contexts were observed in *Yersinia* and *Pseudomonas* genomes, as well as downstream of an *hcp-vgrG* operon in *Shewanella algae*, and downstream of one of multiple *vgrG* paralogs in *Cronobacter dublinensis* (Figure 5B). In some genomes, the modules were also positioned adjacent to additional Rhs toxin–immunity pairs, consistent with polymorphic toxin cassette expansion [5].

In addition to T6SS-associated regions, both module types were identified in heterogeneous genetic neighborhoods. For YPO3609CT-YPO3610, representatives included regions downstream of a toxin–antitoxin system (Figure 5A) [49] and regions containing transposases, phage-associated regulatory elements, or tRNA loci. For YPO3615CT-YPO3616, representative examples included residing in a chaperone-usher (CU) fimbrial gene island or an integrative and conjugative element (ICE)-like region (Figure 5B). These representative examples illustrate the variable genomic contexts of the two module types.

## 4. Discussion

In this study, we functionally characterized two Rhs nuclease toxin–immunity modules, YPO3609-YPO3610 and YPO3615-YPO3616, in *Y. pestis*. Both toxins harbor a conserved Rhs domain followed by a variable C-terminal nuclease domain. YPO3609 exerts nuclease-associated antibacterial activity mediated by its C-terminal WHH domain (residues 374–421) [41], with H411 acting as a conserved putative active-site residue, as supported by the conservation of H411 within the WHH motif [42]. Meanwhile, YPO3615 mediates bacterial killing through the nuclease-associated activity of its C-terminal restriction endonuclease-like domain (residues 1382–1512), with D1442 and D1447 contributing as putative functional residues [43]. Mutations at these putative functional residues attenuated nuclease activity, SOS-related response, and associated filamentation, supporting their functional importance. Their cognate immunity proteins, YPO3610 and YPO3616, respectively, encoded by immediately downstream genes, specifically neutralized the toxicity and reduced DNA degradation (Figure 2C,D and Figure 3), consistent with the canonical toxin–immunity pair architecture [10].

Beyond this shared Rhs core and C-terminal nuclease domain, YPO3609 and YPO3615 differ in the conserved PxxxxDPxGL motif, which demarcates the Rhs core from the variable C-terminal toxin domain and has been associated with cleavage-related processing [40,50,51]. YPO3615 carries the canonical motif (P-x_4_-DPxGL), whereas YPO3609 contains a non-canonical variant with an expanded spacer (P-x_9_-DPxGL) (Figure 1A,B). In some T6SS-associated Rhs effectors, autoproteolytic cleavage does not necessarily trigger immediate toxin release; instead, the cleaved toxin can remain sequestered within the Rhs cage, whereas full liberation may require additional conformational changes or mechanical forces during effector delivery [40]. Because this motif is cleavage-associated rather than a simple on/off determinant of cleavage, the biological consequence of the expanded spacer in YPO3609 cannot be inferred directly from sequence alone and remains to be determined experimentally.

The SOS response is a widely conserved bacterial DNA damage response network. Upon DNA damage, RecA assembles on single-stranded DNA (ssDNA) to form nucleoprotein filaments, which promote LexA autoproteolysis and thereby relieve transcriptional repression of SOS genes, including *recA* and *sulA* [44,52]. Both toxin fragments increased *recA* promoter activity (Figure 3D,E), consistent with their nuclease activity. Despite the pronounced DNA degradation observed in the toxin-expressing strains (Figure 3A–C), the *recA* responses were weaker than those induced by the positive-control inducers CIP and MMC and therefore did not indicate strong canonical SOS activation. SOS-related readouts reflect the cellular response to DNA damage rather than directly quantifying the extent of DNA degradation. The lower magnitude relative to the positive controls may also reflect differences in the mode of DNA damage generation [53], as these positive controls are exogenous DNA-damaging agents, whereas YPO3609 and YPO3615 are intracellularly expressed nuclease toxins. The magnitude of the *recA* response was comparable to that reported for another intracellularly expressed bacterial nuclease, the *Salmonella bongori* VRR-Nuc effector TseV2, in *E. coli* [54]. Consistent with these SOS-related changes, *sulA* mRNA levels were increased in strains expressing the WT toxin fragments compared with the corresponding mutants (Figure 4A,B). The gene *sulA* encodes a cell division inhibitor of FtsZ polymerization; its upregulation provides a primary molecular basis for SOS-associated cell-division arrest [46].

Microscopic examination further revealed that WT toxin expression caused pronounced bacterial elongation and filamentous morphology, whereas the corresponding mutants markedly attenuated this phenotype (Figure 4C,D), consistent with DNA damage-associated cell-division defects and the upregulation of *sulA*. Interestingly, we also observed a significant reduction in *ftsZ* mRNA levels in the WT toxin-expressing strains (Figure 4A,B). Given that canonical SOS-associated filamentation is primarily linked to *sulA* upregulation, the reduced *ftsZ* mRNA levels observed here should be interpreted cautiously and may reflect a secondary consequence of DNA damage-associated stress or growth inhibition [47,55]. Indeed, decreased *ftsZ* expression has been shown to increase cell length in *E. coli* [21]. Collectively, these findings indicate that YPO3609 and YPO3615 cause DNA degradation and induce SOS-related transcriptional responses, with *sulA* upregulation as a primary molecular readout. The resulting filamentation phenotype here likely reflects *sulA* upregulation, while a contribution from reduced *ftsZ* expression cannot be excluded.

One limitation should nevertheless be considered when interpreting the attenuated activities observed for the mutant constructs: the protein levels of the WT and mutant toxin fragments were not directly assessed. Although the potential effects of the substitutions on protein stability were evaluated computationally, these predictions cannot substitute for direct protein-level evidence. Accordingly, the attenuated phenotypes of the mutant constructs may reflect not only impairment of the putative functional residues but also altered protein abundance or stability, either of which could have contributed to the observed reductions in growth inhibition, DNA degradation, SOS-related responses, and cell filamentation.

Co-expression of each toxin with its cognate immunity protein neutralized toxicity. YPO3610 protected cells against YPO3609-mediated growth inhibition and filamentation, while YPO3616 provided equivalent protection against YPO3615 (Figure 2C,D and Figure 4C,D). Correspondingly, DNA degradation was absent in cells co-expressing toxin–immunity pairs (Figure 3). These results establish YPO3610 and YPO3616 as specific immunity proteins that confer self-protection against their respective Rhs nuclease toxins, functionally validating the toxin–immunity architecture predicted by gene organization.

Homologs of YPO3609CT and YPO3615CT occur across multiple bacterial taxa, predominantly within Pseudomonadota and class Gammaproteobacteria, with YPO3609CT showing broader distribution (Figure A1 and Figure A2). In many genomes, the YPO3609CT-YPO3610 or YPO3615CT-YPO3616 toxin–immunity modules are located within T6SS gene clusters or downstream of *hcp/vgrG* (Figure 5A,B), indicating that these modules often occur in T6SS-associated genomic contexts. Some modules are positioned adjacent to additional toxin–immunity modules, consistent with the cassette-like organization typical of polymorphic toxin systems [5]. However, their genomic contexts extend beyond T6SS-associated loci. Some modules occur with CU fimbrial gene islands or adhesion-related structural genes, suggesting that these modules can be embedded in genomic islands with distinct functional features [56]. In other contexts, they are associated with transposases, phage-associated regulatory elements, tRNA loci, or ICE-like regions, suggesting they may have experienced integration and recombination [57,58]. Collectively, these observations show that the two toxin–immunity modules are not restricted to T6SS-associated loci, but also occur in heterogeneous genetic neighborhoods.

## 5. Conclusions

This study functionally characterizes YPO3609-YPO3610 and YPO3615-YPO3616 as two nuclease-active Rhs toxin–immunity modules encoded by *Y. pestis*. When heterologously expressed in *E. coli*, the C-terminal WHH domain of YPO3609 and the restriction endonuclease-like domain of YPO3615 mediate DNA degradation, induce SOS-related responses, and cause cell-division defects, with H411 acting as a conserved putative active-site residue in YPO3609 and D1442/D1447 contributing as putative functional residues in YPO3615. Their cognate immunity proteins, YPO3610 and YPO3616, confer specific self-protection by neutralizing nuclease toxicity. Comparative genomic analysis further showed that these modules and their related homologs occur primarily within Pseudomonadota and are found in both T6SS-associated loci and heterogeneous genetic neighborhoods. Together, these findings provide a functional characterization of *Y. pestis*-encoded Rhs toxin–immunity modules and add to our understanding of Rhs-associated nuclease toxins and their related homologs.

## Data Availability

The original contributions presented in this study are included in the article. Further inquiries can be directed to the corresponding author.

## Appendix A

**Table A1:** Strains and plasmids used in this study.

Strains	Description
*Y. pestis*	
EV76	*pgm-*
*E. coli*	
DH5α	K-12 F*^-^* λ*^-^ recA1 lacZΔM15 thi-*1
MG1655	K-12 F*^-^* λ*^-^ ilvG^-^ rfb-50 rph-1*
BL21(DE3)	F*^-^ ompT hsdSB*(rB- mB-) *gal dcm* λ(DE3)
**Plasmids**	**Description**
pBAD24	Cloning vector with *araBAD* promoter, ColE1 *ori*, *araC*, Amp^R^
pBAD24-YPO3609^306–456^ *	YPO3609^306–456^ cloned at *EcoR*I/*Hind*III sites in pBAD24
pBAD24-YPO3609^306–456(H411A)^ *	H411A mutant of YPO3609^306–456^ in pBAD24
pBAD24-YPO3616 *	YPO3616 cloned at *EcoR*I/*Hind*III sites in pBAD24
pBAD33	Cloning vector with *araBAD* promoter, p15A *ori*, *araC*, Cm^R^, engineered SD sequence
pBAD33-YPO3610 *	YPO3610 cloned at *Kpn*I/*Hind*III sites in pBAD33
pBAD33-YPO3615^1382–1512^ *	YPO3615^1382–1512^ cloned at *Kpn*I/*Hind*III sites in pBAD33
pBAD33-YPO3615^1382–1512(D1442N, D1447N)^ *	D1442N and D1447N double mutant of YPO3615^1382–1512^ in pBAD33
pBAD24-YPO3615^1382–1512^ *	YPO3615^1382–1512^ cloned at *EcoR*I/*Hind*III sites in pBAD24
pBAD24-YPO3615^1382–1512(D1442N, D1447N)^ *	D1442N and D1447N double mutant of YPO3615^1382–1512^ in pBAD24
pBBR*lux*	Plasmid containing a promoterless *lux* reporter, Cm^R^
pBBR*lux-recA* *	*recA* promoter region of *Y. pestis* cloned at *Sac*I/*BamH*I in pBBR*lux*

* Recombinant plasmids constructed in this study.

**Table A2:** Primers used in this study.

Primer	Oligonucleotide Sequence (5′-3′) ^a^	Target
EcoRI-3609CT-p24F	ttgggctagcaggaggaattcACCATGGCGGGGGGGATA	pBAD24-ypo3609^306–456^
EV3609CT-F2dn	tccgccaaaacagccaagcttTTAACACCCCGACCTTAGCG	
EV3609CT-H411A-F1dn	GAAACTGAACCCGCAGCAGGGAATGTAGCTTC	pBAD24-ypo3609^306–456(H411A)^
EV3609CT-H411A-F2up	GAAGCTACATTCCCTGCTGCGGGTTCAGTTTC	
KpnI-3610-pGHF	ggaggaattcaccatggtaccCAAAAATCCTTGTCAGAATTGTGAG	pBAD33-ypo3610
HindIII-3610-pGHR	tccgccaaaacagccaagcttTTACTCTAAATCGTCTTCTTCGACCA	
KpnI-3615(1382)-p33F	ggaggaattcaccatggtaccCATGCGTGAAGTTAACGGAACA	pBAD33-ypo3615^1382–1512^
3615CT-F2dn	caaaacagccaagcttCTATATACCAGAAATAAG	
3615CT-DD-F1dn	CATTCATATTAACAGCCATAATATTAGGTCGTC	pBAD33-ypo3615^1382–1512(D1442N, D1447N)^
3615CT-DD-F2up	GACGACCTAATATTATGGCTGTTAATATGAATG	
EcoRI-3616-p24F	ttgggctagcaggaggaattcACCATGTGTACGGCTATAGCTATTTAT	pBAD24-ypo3616
HindIII-3616-p24R	tccgccaaaacagccaagcttTTATGGCAGTTTTCTAGGTGCTTT	
EcoRI-3615(1382)-p24F	ttgggctagcaggaggaattcACCATGCGTGAAGTTAACGGA	pBAD24-ypo3615^1382–1512^, pBAD24-ypo3615^1382–1512(D1442N, D1447N)^
PrecA500-SacI-F	ctatagggcgaattggagctcCCAAAGCCCTGACGGATATTG	pBBR*lux-recA*
PrecA-BamHI-rev	gcggccgcaactagaggatccCATTTTTATTCCCTCTCTGGTATGC	
*rpoD*-fw	ACGCCGATCGGTGATGATGAAGAT	RT-qPCR
*rpoD*-rev	TCGATACCGAAACGCATACGCAGA	
*sulA*-fw	GCCGGGCTTATCAGTGAAGT	
*sulA*-rev	CCTGAACCCATTCCCGACTC	
*ftsZ*-F	CTTCTCTTGACCCGGATATG	
*ftsZ*-R	CATTCACGACTTTAGCAACC	

^a^ Lowercase letters indicate nucleotides complementary to the sequences of cloning vectors.

## References

[1] Perry R.D., Fetherston J.D. (1997). *Yersinia pestis*—Etiologic agent of plague. Clin. Microbiol. Rev..

[2] Demeure C.E., Dussurget O., Mas Fiol G., Le Guern A.-S., Savin C., Pizarro-Cerdá J. (2019). *Yersinia pestis* and plague: An updated view on evolution, virulence determinants, immune subversion, vaccination, and diagnostics. Genes. Immun..

[3] Chouikha I., Hinnebusch B.J. (2012). *Yersinia*—flea interactions and the evolution of the arthropod-borne transmission route of plague. Curr. Opin. Microbiol..

[4] Price S.L., Vadyvaloo V., DeMarco J.K., Brady A., Gray P.A., Kehl-Fie T.E., Garneau-Tsodikova S., Perry R.D., Lawrenz M.B. (2021). Yersiniabactin contributes to overcoming zinc restriction during Yersinia pestis infection of mammalian and insect hosts. Proc. Natl. Acad. Sci. USA.

[5] Jamet A., Nassif X. (2015). New players in the toxin field: Polymorphic toxin systems in bacteria. mBio.

[6] Cascales E., Buchanan S.K., Duché D., Kleanthous C., Lloubès R., Postle K., Riley M., Slatin S., Cavard D. (2007). Colicin biology. Microbiol. Mol. Biol. Rev..

[7] Aoki S.K., Pamma R., Hernday A.D., Bickham J.E., Braaten B.A., Low D.A. (2005). Contact-dependent inhibition of growth in *Escherichia coli*. Science.

[8] Koskiniemi S., Lamoureux J.G., Nikolakakis K.C., t’Kint de Roodenbeke C., Kaplan M.D., Low D.A., Hayes C.S. (2013). Rhs proteins from diverse bacteria mediate intercellular competition. Proc. Natl. Acad. Sci. USA.

[9] Ruhe Z.C., Low D.A., Hayes C.S. (2020). Polymorphic toxins and their immunity proteins: Diversity, evolution, and mechanisms of delivery. Annu. Rev. Microbiol..

[10] Jackson A.P., Thomas G.H., Parkhill J., Thomson N.R. (2009). Evolutionary diversification of an ancient gene family (rhs) through C-terminal displacement. BMC Genom..

[11] Poole S.J., Diner E.J., Aoki S.K., Braaten B.A., t’Kint de Roodenbeke C., Low D.A., Hayes C.S. (2011). Identification of functional toxin/immunity genes linked to contact-dependent growth inhibition (CDI) and rearrangement hotspot (Rhs) systems. PLoS Genet..

[12] Jurėnas D., Payelleville A., Roghanian M., Turnbull K.J., Givaudan A., Brillard J., Hauryliuk V., Cascales E. (2021). Photorhabdus antibacterial Rhs polymorphic toxin inhibits translation through ADP-ribosylation of 23S ribosomal RNA. Nucleic Acids Res..

[13] Deng W., Burland V., Plunkett G., Boutin A., Mayhew G.F., Liss P., Perna N.T., Rose D.J., Mau B., Zhou S. (2002). Genome sequence of *Yersinia pestis* KIM. J. Bacteriol..

[14] Berger M., Aijaz I., Berger P., Dobrindt U., Koudelka G. (2019). Transcriptional and Translational Inhibitors Block SOS Response and Shiga Toxin Expression in Enterohemorrhagic Escherichia coli. Sci. Rep..

[15] Maslowska K.H., Makiela-Dzbenska K., Fijalkowska I.J., Schaaper R.M. (2015). Suppression of the *E. coli* SOS response by dNTP pool changes. Nucleic Acids Res..

[16] Wu B., Liang W., Kan B. (2015). Enumeration of viable non-culturable *Vibrio cholerae* using propidium monoazide combined with quantitative PCR. J. Microbiol. Methods.

[17] Charbonneau M.E., Mourez M. (2008). The *Escherichia coli* AIDA-I autotransporter undergoes cytoplasmic glycosylation independently of export. Res. Microbiol..

[18] Kurasz J.E., Hartman C.E., Samuels D.J., Mohanty B.K., Deleveaux A., Mrázek J., Karls A.C. (2018). Genotoxic, Metabolic, and Oxidative Stresses Regulate the RNA Repair Operon of Salmonella enterica Serovar Typhimurium. J. Bacteriol..

[19] Felício D.F., Vidal Lda S., Irineu R.S., Leitão A.C., von Kruger W.A., Britto Cde P., Cardoso A., Cardoso J.S., Lage C. (2013). Overexpression of Escherichia coli nucleotide excision repair genes after cisplatin-induced damage. DNA Repair.

[20] Feliciello I., Đermić E., Malović H., Ivanković S., Zahradka D., Ljubić S., Procino A., Đermić D. (2022). Regulation of ssb Gene Expression in Escherichia coli. Int. J. Mol. Sci..

[21] Zheng H., Ho P.Y., Jiang M., Tang B., Liu W., Li D., Yu X., Kleckner N.E., Amir A., Liu C. (2016). Interrogating the Escherichia coli cell cycle by cell dimension perturbations. Proc. Natl. Acad. Sci. USA.

[22] Marchler-Bauer A., Lu S., Anderson J.B., Chitsaz F., Derbyshire M.K., DeWeese-Scott C., Fong J.H., Geer L.Y., Geer R.C., Gonzales N.R. (2010). CDD: A Conserved Domain Database for the functional annotation of proteins. Nucleic Acids Res..

[23] Gabler F., Nam S.Z., Till S., Mirdita M., Steinegger M., Söding J., Lupas A.N., Alva V. (2020). Protein sequence analysis using the MPI bioinformatics toolkit. Curr. Protoc. Bioinform..

[24] Kumar S., Stecher G., Tamura K. (2016). MEGA7: Molecular evolutionary genetics analysis version 7.0 for bigger datasets. Mol. Biol. Evol..

[25] Crooks G.E., Hon G., Chandonia J.-M., Brenner S.E. (2004). WebLogo: A sequence logo generator. Genome Res..

[26] Xie Y., Li H., Luo X., Li H., Gao Q., Zhang L., Teng Y., Zhao Q., Zuo Z., Ren J. (2022). IBS 2.0: An upgraded illustrator for the visualization of biological sequences. Nucleic Acids Res..

[27] Pires D.E., Ascher D.B., Blundell T.L. (2014). DUET: A server for predicting effects of mutations on protein stability using an integrated computational approach. Nucleic Acids Res..

[28] Rodrigues C.H.M., Pires D.E.V., Ascher D.B. (2021). DynaMut2: Assessing changes in stability and flexibility upon single and multiple point missense mutations. Protein Sci..

[29] Camacho C., Coulouris G., Avagyan V., Ma N., Papadopoulos J., Bealer K., Madden T.L. (2009). BLAST+: Architecture and applications. BMC Bioinform..

[30] Cox E., Tsuchiya M.T., Ciufo S., Torcivia J., Falk R., Anderson W.R., Holmes J.B., Hem V., Breen L., Davis E. (2025). NCBI taxonomy: Enhanced access via NCBI datasets. Nucleic Acids Res..

[31] Eddy S.R. (2011). Accelerated Profile HMM Searches. PLoS Comput. Biol..

[32] Eddy S.R. (1998). Profile hidden Markov models. Bioinformatics.

[33] Bateman A., Coin L., Durbin R., Finn R.D., Hollich V., Griffiths-Jones S., Khanna A., Marshall M., Moxon S., Sonnhammer E.L. (2004). The Pfam protein families database. Nucleic Acids Res..

[34] Haft D.H., Selengut J.D., Richter R.A., Harkins D., Basu M.K., Beck E. (2012). TIGRFAMs and genome properties in 2013. Nucleic Acids Res..

[35] Shen W., Le S., Li Y., Hu F. (2016). SeqKit: A Cross-Platform and Ultrafast Toolkit for FASTA/Q File Manipulation. PLoS ONE.

[36] Schwengers O., Jelonek L., Dieckmann M.A., Beyvers S., Blom J., Goesmann A. (2021). Bakta: Rapid and standardized annotation of bacterial genomes via alignment-free sequence identification. Microb. Genom..

[37] Gilchrist C.L.M., Chooi Y.H. (2021). clinker & clustermap.js: Automatic generation of gene cluster comparison figures. Bioinformatics.

[38] Yang X., Pan J., Wang Y., Shen X. (2018). Type VI secretion systems present new insights on pathogenic Yersinia. Front. Cell. Infect. Microbiol..

[39] Andersson J.A., Sha J., Erova T.E., Fitts E.C., Ponnusamy D., Kozlova E.V., Kirtley M.L., Chopra A.K. (2017). Identification of New Virulence Factors and Vaccine Candidates for *Yersinia pestis*. Front. Cell Infect. Microbiol..

[40] Günther P., Quentin D., Ahmad S., Sachar K., Gatsogiannis C., Whitney J.C., Raunser S. (2022). Structure of a bacterial Rhs effector exported by the type VI secretion system. PLoS Pathog..

[41] Zhang D., Iyer L.M., Aravind L. (2011). A novel immunity system for bacterial nucleic acid degrading toxins and its recruitment in various eukaryotic and DNA viral systems. Nucleic Acids Res..

[42] Tang L., Dong S., Rasheed N., Wu H.W., Zhou N., Li H., Wang M., Zheng J., He J., Chao W.C.H. (2022). *Vibrio parahaemolyticus* prey targeting requires autoproteolysis-triggered dimerization of the type VI secretion system effector RhsP. Cell Rep..

[43] Tang J.Y., Bullen N.P., Ahmad S., Whitney J.C. (2018). Diverse NADase effector families mediate interbacterial antagonism via the type VI secretion system. J. Biol. Chem..

[44] Little J.W., Mount D.W. (1982). The SOS regulatory system of *Escherichia coli*. Cell.

[45] Huisman O., D’Ari R. (1981). An inducible DNA replication–cell division coupling mechanism in *E. coli*. Nature.

[46] Mukherjee A., Cao C., Lutkenhaus J. (1998). Inhibition of FtsZ polymerization by SulA, an inhibitor of septation in *Escherichia coli*. Proc. Natl. Acad. Sci. USA.

[47] Miguel A., Zietek M., Shi H., Sueki A., Corona F., Maier L., Verheul J., den Blaauwen T., Van Valen D., Typas A. (2025). Modulation of bacterial cell size and growth rate via activation of a cell envelope stress response. mBio.

[48] González-Magaña A., Tascón I., Altuna-Alvarez J., Queralt-Martín M., Colautti J., Velázquez C., Zabala M., Rojas-Palomino J., Cárdenas M., Alcaraz A. (2023). Structural and functional insights into the delivery of a bacterial Rhs pore-forming toxin to the membrane. Nat. Commun..

[49] Hayes F. (2003). Toxins-antitoxins: Plasmid maintenance, programmed cell death, and cell cycle arrest. Science.

[50] Jurėnas D., Rosa L.T., Rey M., Chamot-Rooke J., Fronzes R., Cascales E. (2021). Mounting, structure and autocleavage of a type VI secretion-associated Rhs polymorphic toxin. Nat. Commun..

[51] Hayes B.K., Harper M., Venugopal H., Lewis J.M., Wright A., Lee H.C., Steele J.R., Steer D.L., Schittenhelm R.B., Boyce J.D. (2024). Structure of a Rhs effector clade domain provides mechanistic insights into type VI secretion system toxin delivery. Nat. Commun..

[52] Maslowska K.H., Makiela-Dzbenska K., Fijalkowska I.J. (2019). The SOS system: A complex and tightly regulated response to DNA damage. Environ. Mol. Mutagen..

[53] Zeynep Baharoglu D.M. (2014). SOS, the formidable strategy of bacteria against aggressions. FEMS Microbiol. Rev..

[54] Hespanhol J.T., Sanchez-Limache D.E., Nicastro G.G., Mead L., Llontop E.E., Chagas-Santos G., Farah C.S., de Souza R.F., Galhardo R.D.S., Lovering A.L. (2022). Antibacterial T6SS effectors with a VRR-Nuc domain are structure-specific nucleases. eLife.

[55] Vicente M., Gomez M.J., Ayala J.A. (1998). Regulation of transcription of cell division genes in the *Escherichia coli* dcw cluster. Cell Mol. Life Sci..

[56] Werneburg G.T., Thanassi D.G. (2018). Pili Assembled by the Chaperone/Usher Pathway in *Escherichia coli* and *Salmonella*. EcoSal Plus.

[57] Delavat F., Miyazaki R., Carraro N., Pradervand N., van der Meer J.R. (2017). The hidden life of integrative and conjugative elements. FEMS Microbiol. Rev..

[58] Juhas M., van der Meer J.R., Gaillard M., Harding R.M., Hood D.W., Crook D.W. (2009). Genomic islands: Tools of bacterial horizontal gene transfer and evolution. FEMS Microbiol. Rev..

